# Quantitative Changes in Various Nutrient Ratios in Fodder Plants as an Effect of Compost and Fly Ash Application

**DOI:** 10.3390/ijerph19138136

**Published:** 2022-07-02

**Authors:** Monika Jakubus, Małgorzata Graczyk

**Affiliations:** 1 Department of Soil Science and Microbiology, Poznan University of Life Sciences, 60-656 Poznań, Poland; 2 Department of Mathematical and Statistical Methods, Poznan University of Life Sciences, 60-656 Poznań, Poland; malgorzata.graczyk@up.poznan.pl

**Keywords:** immobilizing agents oat, lupine, macroelements, soil contaminated with Cu

## Abstract

Despite the popularity of concentrated feed, fodder crops are still important, especially in organic livestock farming. However, this type of feed must meet certain criteria, which are often described using correct nutrient ratios. The research investigates the influence of compost and fly ash on quantitative changes in nutrient ratios determined for lupine and oat cultivated on soil slightly contaminated with Cu. A pot experiment was conducted on medium soil. Immobilizing agents (compost and fly ash) were applied at the dose of 40 t per ha. Plant materials were subjected to chemical analyses to assess their macronutrient content and, based on these data, mutual proportions of individual nutrients were calculated as mass ratios of K:Mg, K:Na, Ca:P, Ca:Mg, K:(Ca+Mg) and (K+Na):(Ca+Mg). Changes in ratio values were visualized using statistical tools, i.e., Anova, correlation coefficients and dendrograms. It was found that immobilizing agents constituted a source of the chosen nutrients because their amounts in plants grown on the soil fertilized with either compost or fly ash compost were significantly higher than in the control plants. This phenomenon was especially visible with regard to Ca and Mg for both lupine and oat. It should be emphasized here that the effect of compost or fly ash on the content of Ca and K in plants was comparable. In general, the application of compost contributed to higher values of the analyzed nutrients in both plants. The control and fertilized fly ash plants were characterized by lower values of nutrient ratios. The expected optimal value range of 2–3:1 was determined only for Ca:Mg, which was found in both lupine and oats. The proper values of K:Mg (2–6:1) were found only in the case of fodder plants cultivated on soil amended with compost.

## 1. Introduction

Despite the rise in concentrate feeding, forage crops are still widely used as the main source of feed to its high–yields of DM [1]. Cited authors indicated that the group of forage crops comprise great varieties of plants; however, the grasses and legumes prevail. Especially lupins are very important in the animal diet because sweet lupin, e.g., narrow-leaved one, is rich in protein (32%). Lupins are also characterized by a high content of carotenoids. Narrow-leaved lupine is a popular legume species because of its high yielding potential, increased thermal tolerance and shorter vegetation period. This species is a valuable protein source and may be used as fodder in the form of seeds, green forage and silage [2].

Oat is also recognized as a significant fodder crop, widely grown for green fodder as well as grain in different parts of the world. It ranks sixth in the world cereal production following wheat, maize, rice, barley and sorgum [3]. According to Dangi [4], oat can be cultivated in combination with other fodder legumes such as lucerne, pea and vetch. Oat is characterized by high yields and suitability for cultivation in a wide range of soil types and climatic conditions. It influences the high attractiveness of oats, especially at the early harvest in the form of green fodder, because it ensures better growth than legumes and grasses cultivated as intersowns [5]. Additionally, the green forage of oats is a good source of protein, fiber and minerals. It is used as fresh fodder, feed and straw, but can also be used as silage/hay during fodder deficit periods. According to Kumar et al. [6], green fodder is relished by all animals because of its higher palatability and softness than that of wheat and barley.

Due to fact that green fodder plays a major role in the natural feed of animals, and the quality of animal products (meat, dairy, eggs) is considerably determined by the nutritional value of forage, their evaluation of quality is necessary and mandatory. In this context, usually, the content of macro and micronutrients essential for plants is taken into account. However, it should be also considered that each crop has different requirements for nutrients and their accumulation in tissues is changeable and mainly depends on the vegetation stage. Taking this fact into account, Kumar and Soni [7] indicated that not only optimal amounts of nutrients but also their proper balance is significant for proper plant development. So more the mutual nutrient ratios have specific values or their ranges are constant for plants, regardless of the plant’s nutritional needs and differences between species. Moreover, the proportions and relationship between macronutrients in plant biomass can be indicators of vegetation composition and nutrient limitation [8]. However, the assessment of nutrient ratios is less popular. Besides, the authors present nutrient ratios in various ways as in the literature one can find the ionic, mass and even molar ratios of nutrients [8,9,10,11,12]. Macro- and micronutrient contents and their mutual relationships in crops result from, among other things, nutrient availability for plant roots and nutrient antagonisms and synergisms phenomenon. The importance of the nutrient antagonisms and synergism effects on yield and fertilizer use efficiency was underlined by Rietra et al. [13]. Thus, this aspect becomes more important when cultivating fodder crops on soils with less favorable parameters, including those related to reduced nutrient content, unregulated soil pH, unfavorable moisture conditions or increased content of heavy metals. All the above-mentioned factors, apart from the presence of heavy metals, can be eliminated through good agricultural practices related to proper liming, fertilization and irrigation. Generally, the presence of heavy metals in soil is an unfavorable factor, because heavy metal accumulation in agricultural soils leads to lower nutrient availability to plants and deterioration of soil functions that have a great impact on the production and quality of crops [6]. In the case of the content of heavy metals in soil, the most important criterion is their total content, which enables the determination of the degree of heavy metal contamination, and thus allows or excludes the agricultural use of the land. Soils with a slightly increased content of heavy metals that are also essential micronutrients for plants, such as Cu and Zn, can be used for agricultural purposes, excluding the cultivation of plants directly consumed by humans. At the same time, in such cases, remediation methods should be used based on chemical immobilization techniques. As reported by Awasthi et al. [14], those are among the most common and effective methods. The immobilization process is based on a reduction in the bioavailability of metals, which leads to decreasing mobility and limitation of the metals uptake by plants. In practice, usually organic (compost, manure, biochar) and inorganic (fly ash, lime, natural zeolites) substance are used as immobilization agents [15]. Using organic matter or alkalizing substances leads to the creation of insoluble organic-metallic or metal-mineral forms, which directly inactivates heavy metals in the soil and reduces their potential negative accumulation in plant biomass. However, as previously mentioned, some heavy metals are also essential micronutrients for plants and both their excess and deficiency can be harmful to plants, leading to disorders in proper development, yielding and disease resistance [16]. In the present research, soil with an increased amount of Cu was used in the experiment. Copper deficiency is unfavorable for plants because it is required for reproductive growth, proper development, complete root metabolism and utilization of essential proteins. Despite this, the excess of Cu was found to be extremely toxic and may have a morphological, physiological, biochemical and phytotoxic effect [16]. The use of immobilizing agents in soils that require remediation is entirely justified, however, one should be aware of their wider impact, for example on the physicochemical and chemical properties of soils. Thus, the introduced substances can immobilize not only metal (Cu in this study) but also the bioavailability of macronutrients that can simultaneously modify the uptake of macronutrients, their amount and mutual ratios in plant biomass. The direction and intensity of these changes will depend on many factors, with the chemical nature of immobilizing agents and plant physiology being the decisive factors. Taking into account the speed and ease of incorporation of heavy metals into the chain, knowledge on this subject becomes important for the practice and maintenance of food safety. It should be taken into account that the quality of fodder plants indirectly affects the health of the animals fed with such fodders, which is consistently reflected in the quality of animal products that will be part of the human diet. Therefore, the research goals are connected with the effect of immobilizing agents (compost and fly ash) on: 1. macronutrient (P, K, Ca, Mg, Na) amounts in fodder plants (lupine and oat) biomass, 2. values of mutual mass ratios between nutrients, 3. potential effect of Cu total content in soil on macronutrient contents and their ratios in plant biomass.

## 2. Materials and Methods

### 2.1. Experimental Design

This study presents a part of a long-term experiment conducted on medium soil (clay loam) classified as *Haplic Cambisol* according to WRB [17]. This soil comes from the area contaminated with copper from the local metallurgical plant. The soil samples for the experiment were taken from the topsoil of the soil in the same separated area, which ensure homogeneity of the material and the same level of metals in the soil. According to Directive [18], the permissible contents for Cu in the soil is up to 140 mg·kg^−1^, while in the soil concerned the amount of copper was determined as 200 mg·kg^−1^, which indicated slightly elevated Cu content. To apply chemical immobilization of Cu in the soil, compost (a mixture of biowaste and manure in a 1:1 ratio, prepared by a specialized composting plant for commercial purposes) was used as organic matter and fly ash (FA—a by-product of lignite combustion) as an alkaline substance.

Table 1 presents the basic properties of soil, compost and fly ash used in this study. Therefore, the design of the experiment included 3 treatments: T0—control soil (without compost or fly ash added), TI—soil with the compost added, TII—soil with the fly ash added, each performed in eight replications. Subsequently, the model of the experiment is expressed in the form $y_{i}^{k}=\mu_{i}^{k}+\alpha_{ij}^{k}+\epsilon_{ij}^{k}$, where $y_{i}^{k}$ denotes the content of *k*^th^ macronutrient in *i*^th^ plant, $\mu_{i}^{k}$ is the general mean of the content of *k*^th^ macronutrient in *i*^th^ plant, $\alpha_{ij}^{k}$ is the effect of *j*^th^ soil on the content of *k*^th^ macronutrient in *i*^th^ plant and $\epsilon_{ij}^{k}\;$ is random error of the content of *k*^th^ macronutrient in *i*^th^ plant growing on *j*^th^ soil  , k∈{P, K, Ca, Mg, Na}, i∈{Lupin, Oat}, $j\in \left\{T_{0},{\;T}_{I},T_{\mathrm{II}}\right\}$. The experiment was treated as the completely random design.

The compost and fly ash were applied into the soil as dry matter substances at an equivalent amount (properly converted into kilograms of soil per hectare, so as to recreate the natural conditions) of 40 t·ha^−1^ two weeks before plant cultivation. The individual substances were thoroughly mixed with soil in separate containers and then transferred to PVC pots (10 kg) and wetted to 60% of field capacity.

Among the crops grown in the crop rotation adopted in the experiment, narrow-leaved lupine (*Lupinus angustifolius* L.) and oats (*Avena sativa* L.) were used as test pasture crops. Both plants at a density of 10 plants per pot were cultivated year by year in crop rotation in the same pot. After harvesting each plant from the pots, soil samples were taken for analysis. To keep the same growing conditions and maintain theoretically the same level of Cu for the next plant in crop rotation, the residue in the pots was cleaned of the remaining roots and the soil, and compost and fly ash were replenished. Taking into account the nutritional requirements of plants, adequate supplementing mineral fertilization was applied. The applied doses of fertilizers were balanced to account for the amounts of N, P, K introduced with compost and fly ash. The fertilizer doses covered the nutritional needs of cultivated plants, which per hectare was 60 kg N, 50 kg P and 60 kg K for oats and 60 kg P and 80 kg K for lupine. For this purpose, ammonium nitrate, triple superphosphate and potassium salt were used. The experiment was conducted outdoor and natural conditions were provided for the experimental facility as it was covered with wire mesh.

### 2.2. Analysis of Plant and Soil

Plant material was dried at 60 °C, ground and ashed in a furnace at 450 °C for 6 h. The ash was dissolved in 5 mL of 6 mol dm^3^ HCl [20] and diluted to a constant volume with distilled water. The obtained extracts were undergone to assessment of K, Ca, Mg, Na contents using atomic absorption spectrophotometry (AAS) in a Varian Spectra AA 220 FS apparatus. Total phosphorus (P) content was measured colorimetrically using the vanadium-molybdenum method. All the assays identifying the amounts of nutrients in the tested samples were performed in three replications. Based on the obtained amounts, the following mass nutrient ratios were calculated: K:Mg, K:Na, Ca:P, Ca:Mg, K:(Ca+Mg) and (K+Na):(Ca+Mg). The selection of the listed nutrient ratios was purposeful and related to the literature reports; those ratios are considered useful parameters in the assessment of crop quality and some of them are used obligatorily in routine chemical tests for agricultural purposes.

### 2.3. Statistical Analysis

To compare the content of macroelements in plants growing on control soil and soil with amendments, a one-way analysis of variance was used after the Shapiro-Wilk test of normality Scheffe (1999) [21]. Moreover, for this analysis, the homogeneous groups were determined. The same method was applied to analyse relationships between the ratios of elements in the plants. Moreover, the dependencies between the ratios were shown on dendrograms. Relationships between the content of Cu in soil and the content of macroelements in the analyzed plants were determined using the Pearson correlation coefficient in the form of heatmaps and presented in the form of regression equalities. The data were analyzed statistically with the R software package at a significance level of α=0.05.

## 3. Results

### 3.1. Lupine

**Figure 1:**
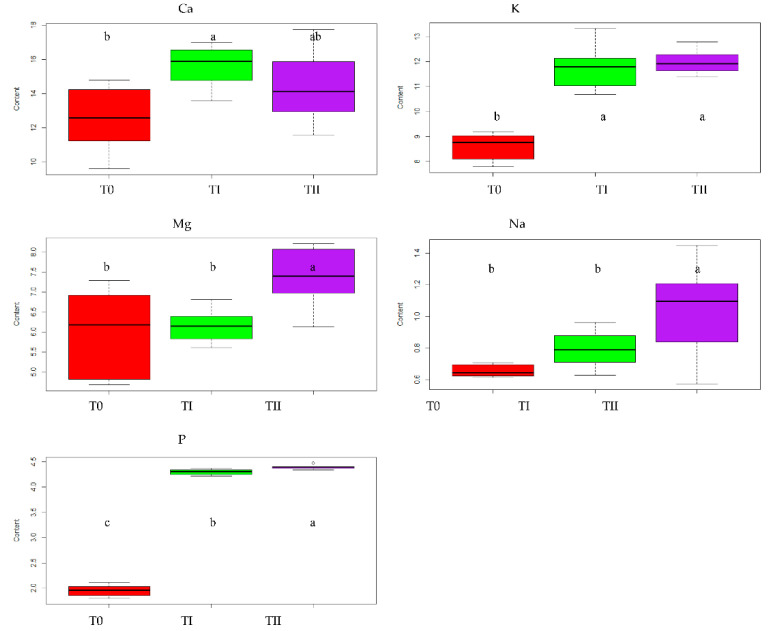
The effect of treating the soil with two amendments on mean Ca, K, Mg, Na and P amounts in lupine grown on soils: T0—control soil, TI—soil with compost addition and TII—soil with FA addition. In the figure, the letters denote homogenous groups.

The lowest contents of all macronutrients were recorded for lupine grown in the control soil: 12.56$\;g\cdot {\mathrm{kg}}^{-1}$ for Ca, 8.7 $g\cdot {\mathrm{kg}}^{-1}\;$ for K, 5.98 $g\cdot {\mathrm{kg}}^{-1}$ for Mg, 0.66 $g\cdot {\mathrm{kg}}^{-1}\;$ for Na and 1.94 $g\cdot {\mathrm{kg}}^{-1}$ for P. The highest nutrient amounts were found in lupine grown in FA enriched soil: 11.96 $g\cdot {\mathrm{kg}}^{-1}\;$ for K, 7.40 $g\cdot {\mathrm{kg}}^{-1}$ for Mg, 1.04 $g\cdot {\mathrm{kg}}^{-1}\;$ for Na and 4.39 $g\cdot {\mathrm{kg}}^{-1}$ for P. The exception was Ca amount (15.63 $g\cdot {\mathrm{kg}}^{-1}$), which was the highest in the conditions of soil with compost addition (Figure 1). The content of phosphorus in lupine was significantly influenced by the immobilization agents applied. P amount was significantly higher for the plants grown on soil fertilized with compost (4.30 $g\cdot {\mathrm{kg}}^{-1})$ and FA (4.39 $g\cdot {\mathrm{kg}}^{-1})\;$ compared to plants grown in control conditions (1.95$\;g\cdot {\mathrm{kg}}^{-1})$ (Table 2, Figure 1). There were no significant differences between the plants grown under control conditions and in compost-enriched soil in terms of Mg and Na contents. On the other hand, the FA application significantly increased the content of those elements in lupine as compared to the content in plants cultivated on soil with other amendments. The average content of magnesium and sodium in plants grown on soil with FA addition was 7.40 $g\cdot {\mathrm{kg}}^{-1}\;$ and 1.04 $g\cdot {\mathrm{kg}}^{-1}$, respectively (Table 2, Figure 1).

The increase in potassium content in lupine was significantly influenced by the enrichment of the soil with immobilizing agents regardless of the type used. A significant difference was noted between the potassium content in plants grown on soil with FA addition (11.96 $g\cdot {\mathrm{kg}}^{-1})$ and the potassium content in plants grown on control soil (8.7 $g\cdot {\mathrm{kg}}^{-1})$ as well between the potassium content in plants grown on soil with compost addition (11.77 $g\cdot {\mathrm{kg}}^{-1})$ and the potassium content in plants grown on control soil (Table 2, Figure 1). Calcium content in lupine cultivated on soil with compost reached an average of 15.63 $g\cdot {\mathrm{kg}}^{-1}$ and was significantly higher than the content of this element in plants grown on control soil 12.56 $g\cdot {\mathrm{kg}}^{-1}$, and at the same time it did not significantly differ from the content in plants grown on soil enriched with FA 14.40 $g\cdot {\mathrm{kg}}^{-1}$.

**Figure 2:**
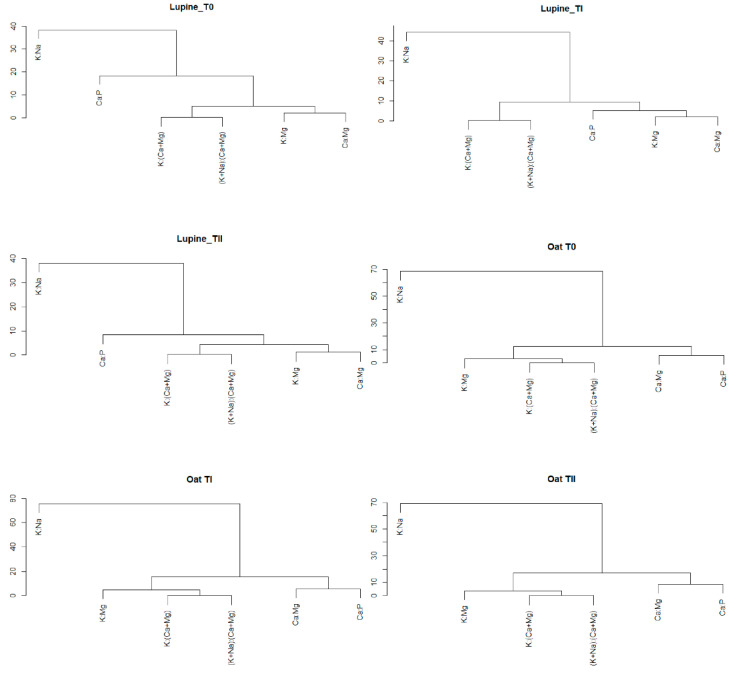
Dendrograms for values Ca:Mg, Ca:P, K:(Ca+Mg), (K+Na):(Ca+Mg), K:Mg and K:Na ratios in lupine and oat grown on soils: T0—control soil, TI—soil with compost addition and TII—soil with FA addition.

**Figure 3:**
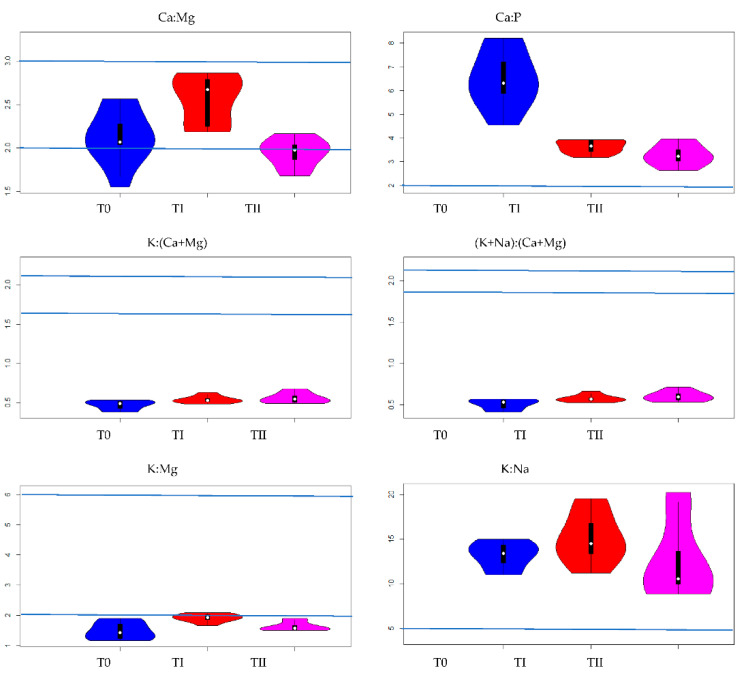
The effect of treating the soil with two amendments on values Ca:Mg, Ca:P, K:(Ca+Mg), (K+Na):(Ca+Mg), K:Mg and K:Na ratios in lupine grown on soils: T0—control soil, TI—soil with compost addition and TII—soil with FA addition. Blue lines denote recommended optimal values of nutrient ratios [8,22].

Among all the analyzed nutrient ratios for lupine, K:Na ratio reached the highest values; regardless of the use of immobilizing agents or their lack, the values varied from 12.6 (TII) to 15.1 (TI). The value of K:Na ratio differed significantly from the values of other nutrient ratios (Figure 2 and Figure 3). At the same time, K:Na value in lupine did not significantly differ among plants grown in different soil conditions (T0–TII).

The values of (K+Na):(Ca+Mg) ratio amounted 0.51 (T0), 0.58 (TI) and 0.60 (TII). At the same time, it can be seen that the value of K:(Ca+Mg) ratio was 0.47, 0.54 and 0.56 for plants grown on T0, TI and TII, respectively (Figure 2 and Figure 3, Table 3). Moreover, the influence of immobilizing agents on the values of the above ratios was similar.

**Figure 4:**
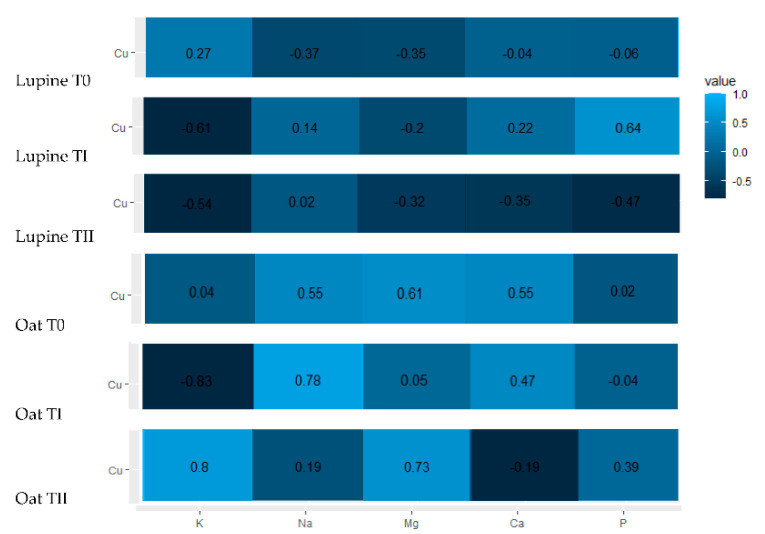
Heatmaps and correlations coefficients for between Cu content in the soils (T0–TII) and nutrients in plants: lupine and oat.

The comparisons of the values of (K+Na):(Ca+Mg) ratios in lupine in different conditions indicated significant differences between the plants grown on control soil and those grown on soil with FA application. The values Ca:Mg, Ca:P, K:(Ca+Mg), (K+Na):(Ca+Mg), K:Mg and K:Na ratios in lupine and oat grown on soils: T0—control soil, TI—soil with compost addition and TII—soil with FA addition are presented on dendrograms. It is worth pointing out, dendrogram is a cluster hierarchy displayed as a tree diagram. It begins with each object in a separate cluster (group) and two clusters that are most similar are joined into a single new cluster. The *y*-axis is a measure of closeness of either individual data points or clusters. The horizontal axis represents the clusters. The vertical scale on the dendrogram represent a measure of closeness of either individual data points or clusters. The vertical position of the split, shown by a short bar gives the distance (dissimilarity) between the two clusters. Under each condition (T0–TII), the analysis of relationships between the values of nutrient ratios in lupine showed similar values for both above-mentioned ratios, (K+Na):(Ca+Mg) and K:(Ca+Mg). The values are presented on dendrograms in the same group for T0, TI and TII and they differ significantly from the others (Figure 3 and Figure 4). In the Figure 3, the point in the middle corresponds to the median, the bold black part in the centre represents interquartile range and the thin black lines represent 1.5 times interquartile.

The value of Ca:Mg ratio amounted to 2.1, 2.6 and 2.0 for plants cultivated on control soil, and soil fertilized with compost and FA, respectively. Significant differences between the values of the aforementioned ratios were observed between plants grown under control conditions and plants grown on soil with compost addition, as well as between plants grown on soil with compost addition and plants grown on soil with FA addition (Figure 3, Table 3). For K:Mg, the following values were obtained: 1.5 (T0), 2.0 (TI) and 1.6 (TII). Significant differences in K:Mg values were observed between plants grown under control conditions and plants grown on soil with compost addition, as well as between plants grown on soil with compost addition and plants grown on soil with FA addition (Figure 3, Table 3). The analysis of relationships between the values of nutrient ratios in lupine indicated that Ca:Mg and K:Mg obtained similar values regardless of experiment factors. They are in the same group on dendrograms for T0, TI and TII and they differed significantly from the others (Figure 2 and Figure 3). The highest value of Ca:P ratio (6.5) was recorded in lupine plants grown on control soil and it was significantly higher than the value of Ca:P ratio in lupine grown on soil with compost addition (3.6) and in lupine grown on soil with FA addition (3.3) (Figure 3, Table 3). The relationships between all ratios presented in Figure 2 indicated that the value of Ca:P ratio was in a separate group in each soil conditions (T0–TII).

It was found that total Cu content in soil was not significantly correlated with the content of macronutrients in lupine (Figure 4). It also had no effect on nutrient ratios.

### 3.2. Oat

**Figure 5:**
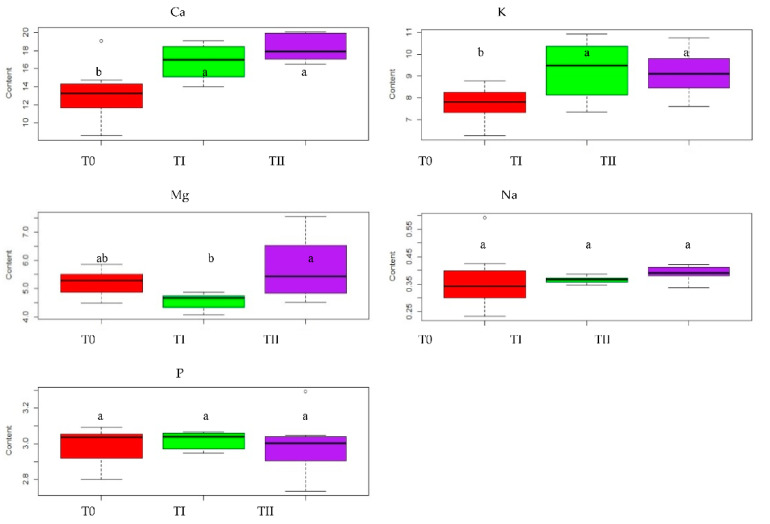
The effect of treating the soil with two amendments on mean Ca, K, Mg, Na and P amounts in oat grown on soils: T0—control soil, TI—soil with compost addition and TII—soil with FA addition. In the figure, the letters denote homogenous groups.

The content of phosphorus and sodium in oat did not differ significantly for control plants and for plants grown on soil enriched with immobilizing agents. The content of phosphorus amounted to 2.99 $g\cdot {\mathrm{kg}}^{-1}$ (T0, TII) and 3.02 $g\cdot {\mathrm{kg}}^{-1}$ (TI) (Table 2, Figure 5). The contents of sodium in plants were very comparable and varied from 0.36 $g\cdot {\mathrm{kg}}^{-1}$ (T0) to 0.39 $g\cdot {\mathrm{kg}}^{-1}$ (TII) (Table 2, Figure 5). In the Figure 5, the bold black line in the box represents the median value of our data, the entire coloured box represents the inter-quartile range, the whiskers represent minimal and maximal values, respectively.

The content of other macronutrients in oat was significantly influenced by the applied immobilizing agents. The content of Ca and K in oat grown on soil enriched with FA or compost was higher than in plants grown on control soil. The content of calcium in oat reached the value of 16.80 $g\cdot {\mathrm{kg}}^{-1}$ in TI and 18.29 $g\cdot {\mathrm{kg}}^{-1}\;$ in TII, which was significantly greater than the content of calcium in control oat, which amounted to 13.27 $g\cdot {\mathrm{kg}}^{-1}$. The content of potassium in oat had a value from 7.71 $g\cdot {\mathrm{kg}}^{-1}\;\left(T0\right)$ to 9.28 $g\cdot {\mathrm{kg}}^{-1}$ (TI) (Table 2, Figure 5). The content of magnesium in oat grown in soil with the addition of FA obtained the value 5.71 $g\cdot {\mathrm{kg}}^{-1}\;$ and was significantly higher than the content of this nutrient in plants grown in soil with the addition of compost (4.55 $g\cdot {\mathrm{kg}}^{-1})$ and in plants grown in control soil (5.21 $g\cdot {\mathrm{kg}}^{-1})\;($Table 2, Figure 5).

**Figure 6:**
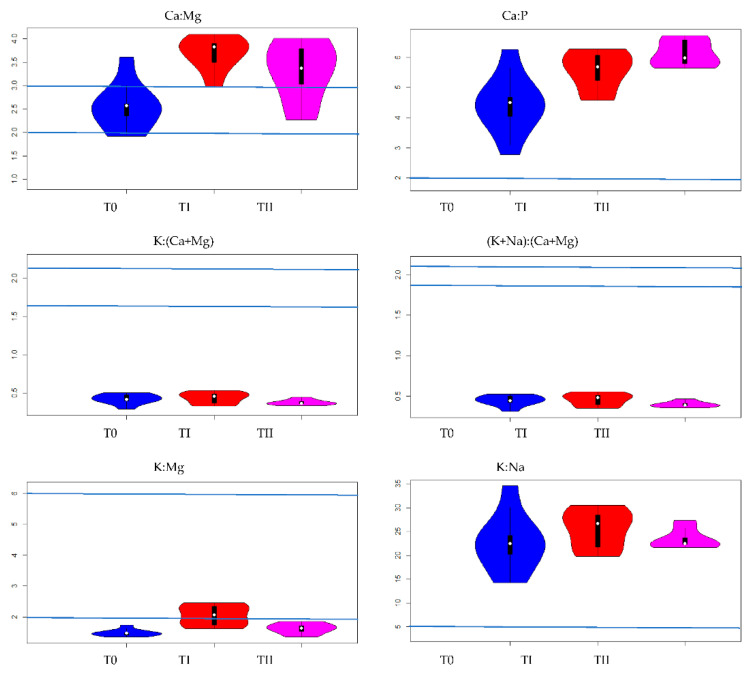
The effect of treating the soil with two amendments on values Ca:Mg, Ca:P, K:(Ca+Mg), (K+Na):(Ca+Mg), K:Mg and K:Na ratios in oat grown on soils: T0—control soil, TI—soil with compost addition and TII—soil with FA addition. Blue lines denote recommended optimal values of nutrient ratios [8,22].

Among all the analyzed nutrient ratios for oat, K:Na ratio reached the highest values regardless of whether the immobilizing agent was used, and its values varied from 22.6 (T0) to 25.4 (TI). The value of K:Na ratio differed significantly from the values of other nutrient ratios (Figure 2 and Figure 6). At the same time, the value of K:Na in oat did not differ significantly among plants grown in different soil conditions (T0–TII). The values of (K+Na):(Ca+Mg) and K:(Ca+Mg) ratios in control plants amounted 0.46 and 0.43, respectively. In plants cultivated on soil amended by compost, the values were 0.44 (K:Ca+Mg)) and 0.46 ((K+Na):(Ca+Mg)). The application of FA resulted in slightly lower values of K:(Ca+Mg) and (K+Na):(Ca+Mg), which amounted to 0.38 and 0.40, respectively (Figure 4, Table 2). The application of immobilizing agents or their lack did not influence the differences between the values of K:(Ca+Mg) in plants grown under experiment conditions (Figure 2 and Figure 6). As was proven above, the values of K:(Ca+Mg) and (K+Na):(Ca+Mg) were very comparable and the influence of experimental factors on these values was not significant. Additionally, the values of those ratios are in the same group on dendrograms for T0, TI and TII and they differed significantly from the others (Figure 2 and Figure 6). For Ca:Mg ratio, the following values were obtained: 2.5 (T0), 3.7 (TI) and 3.3 (TII). Significant differences in Ca:Mg values were observed between plants grown under control conditions and plants grown on soil with compost addition, as well as between plants grown under control conditions and plants grown on soil with FA addition (Figure 6, Table 3). The value of Ca:P ratio amounted to 4.4 (T0), 5.57 (TI) and 6.1 (TII). Significant differences in Ca:P values were observed between plants grown under control conditions and plants growing on soil with compost addition, as well as between plants grown under control conditions and plants grown on soil with FA addition (Figure 6, Table 3). The analysis of relationships between the values of nutrient ratios in oat indicated that the two ratios mentioned above obtain similar values regardless of experiment conditions (T0–TII). Furthermore, these ratios were in the same group on dendrograms individually elaborated for T0, TI and TII and they differed significantly from other ratios (Figure 2 and Figure 6). The following values of K:Mg ratio in oat were obtained: 1.5 (T0), 2.1 (TI) and 1.6 (TII). The value of K:Mg ratio in oat cultivated on soil with compost addition was significantly greater than other values obtained under control conditions and for soil with FA addition. The relationships between all ratios presented in Figure 2 indicated that the value of K:Mg ratio was in a separated group in each soil condition (T0–TII). The content of Cu in the control soil was not significantly correlated with the content of macronutrients in oat. It also had no effect on the ratios of these macronutrients (Figure 4).

The correlation coefficient between Cu content in soil with compost addition and the K amounts was −0.83. It means that an increase in Cu content in soil with compost addition by one gram can lead to a decrease in the K amounts by −0.067 g·kg^−1^ (Figure 4, Table 3 and Table 4). The correlation coefficient between Cu content in soil with compost addition and Na amounts was 0.78, thus an increase in Cu content in soil by one gram can cause an increase by 0.006 g·kg^−1^ in Na amount (Figure 4, Table 3 and Table 4). The Cu content in soil fertilized with FA significantly affected K and Mg amounts in oat, with the values of correlation coefficients of 0.80 and 0.73, respectively (Figure 4, Table 3 and Table 4). Therefore, an increase in Cu content in soil by one gram can cause an increase in K amount by 0.053 g·kg^−1^ and an increase in Mg amount by 0.051 g·kg^−1^ (Figure 4, Table 3 and Table 4). The correlation coefficient between Cu content in control soil and the value of K:Na ratio was −0.72, which means that an increase in Cu content by one gram can lead to a decrease in K:Na value by −0.509 (Figure 4, Table 3 and Table 4).

All significant correlations between Cu content in soil fertilized with compost and nutrient ratios in oat are negative. The correlation coefficient between Cu content in soil and the value of (K+Na):(Ca+Mg) ratio as well as between Cu content in soil and the value of K:(Ca+Mg) ratio was the same: −0.9, which means that an increase in Cu content in soil by one unit can cause a decrease in value (K+Na): (Ca+Mg) ratio by −0.041 and a decrease K:(Ca+Mg) value by −0.042 (Figure 5, Table 3 and Table 4). The value of correlation between Cu content in soil amended by compost and K:Na ratio was equal −0.88 and an increase in Cu content in soil by one unit can cause a decrease in K:Na value in the oat by −0.219. The value of correlation between Cu content in soil (TI) and K:Mg was −0.73, so an increase in Cu content in soil by one unit can lead to a decrease in K:Mg value in the oat by −0.015 (Figure 4, Table 3 and Table 4).

All significant correlations between Cu content in soil fertilized with FA and nutrient ratios in oat are positive. The correlation coefficient between Cu content in soil (TII) and K:Na ratio value was 0.86. It means that an increase in Cu content in soil by one unit can lead to an increase in K:Na value in the oat by 0.174. The correlation coefficient between Cu content in soil and the value of K:(Ca+Mg) ratio was 0.71. Thus, an increase of Cu content in soil by one unit can cause an increase in K:(Ca+Mg) value in the oat by 0.001. A similar relationship was observed for the correlation between Cu in soil and the value of (K+Na):(Ca+Mg) (Figure 4, Table 3 and Table 4).

Figure 4 presents obtained values of nutrient ratios compared to the optimal values indicated in literature and used in practice. The fodder plants should meet quality criteria expressed with correct values of the relationship between macronutrients. The nutrient ratio in the plant should be within a certain optimal range. The ratio values should be 2:1 for Ca:P and 5:1 for K:Na (Figure 6). The obtained values of the above-mentioned ratios exceed these thresholds for both the fodder plants cultivated in three different experimental conditions. At the same time, for both oat and lupine, the values of (K+Na):(Ca+Mg) and K:(Ca+Mg) ratios were lower than the suggested ones, which should be between 1.9–2.1:1 and 1.62–2.2:1, respectively. Analyzing the reference values, it was obtained that $1.9<value\left(\frac{K+Na}{Ca+Mg}\right)<2.1$ and $1.62<value\left(\frac{K}{Ca+Mg}\right)<2.1$ where value(x) means the average ratios of the element x in a given plant and under given experimental conditions. Only for lupine and oat grown on soils enriched with compost, the average value of K:Mg ratio was within the recommended range of 2–6:1. The value of Ca:Mg ratio in plants recommended in the literature is 2–3:1 That criterion was met by both fodder plants regardless of cultivation conditions. Assuming that the average values of Ca:Mg ratio are correct, taking into account the obtained values, it can be concluded that the disturbance in the ratio values is significantly influenced by too low potassium content compared to the content of magnesium, calcium and sodium. If the potassium content was higher, then most likely the macronutrient ratios in both plants would be correct under all experimental conditions.

## 4. Discussion

The main effect of the chemical immobilization process is the reduction of heavy metal bioavailability as a consequence of their conversion into a chemical from which will be inert to biological system and highly insoluble by introducing various sorption process: adsorption to mineral surface precipitation and ion exchange [23]. However, the same effect may be achieved for other elements that are macronutrients. Not only heavy metals can be bound by organic matter but also other elements that are essential nutrients for plants, which is particularly observed in the case of P, Ca, Mg. On the other hand, fly ash as an alkalizing substance may hinder the uptake of phosphorus as a result of the precipitation process of this nutrient in the alkaline environment, forming insoluble salts with calcium. Analyzing the data, it can be concluded that both compost and fly ash were a source of nutrients for the cultivated plants because the lupine and oat grown on fertilized soils either with compost or fly ash had a much higher macronutrient content (the exception was the amount of Na and P in oat, however, the differences were not proved statistically). Regardless of the immobilizing agent and the plant used, the amounts of nutrients decreased in the following series: Ca > K > Mg > P > Na. At this point, it is worth emphasizing that the influence of compost or fly ash on the accumulation of Ca and K levels in both plants was the same, which may be due to the similar amount of these nutrients in compost and fly ash (Table 1). Application of FA caused higher amounts of Na, Mg and P in lupine. The better fertilizing effect of fly ash compared to compost may result from the natural mineralization processes that the organic fertilizers undergo. The mineralization process mainly depends on the soil texture, moisture regime, microbiological activity and the quantity of organic matter incorporated into soil [24,25,26]. Moreover, the decomposition process is more effective and quicker in light soil, where physical properties are more favorable and can accelerate the mineralization process after the application of compost. In this study, the soil used was medium so it can be expected that the mineralization process and its effect in releazing the nutrients shall not be so rapid. For this purpose, some authors [8,9,27,28] indicate a positive aspect of organic fertilizers, while other researchers [12,29] point to a negative influence of such amendments on plant quality.

The quality of fodder plants is largely determined by their nutritional value. It is obligatorily described by the macronutrient contents in plant but balanced proportions between the nutrients are important to ensure proper plant development and yield quality. Moreover, it can be generally assumed that the plant nutrient ratios may provide a better index of deficiency of macronutrients than their concentration [8]. In the literature there are very different and broad ranges of nutrient contents given as an optimal and critical levels for crops [30,31]. Different situation one can notice in relation to the nutrients ratios where values are clearly defined, irrespective of the plants or the fertilization used. Particularly when assessing the fodder nutritive value criteria, correct ratios should be taken into account [10] and the recommended optimal ratios should be as follows: K:Mg = 2–6:1; K:Na = 5:1; Ca:P = 2:1; Ca:Mg = 2–3:1; K:(Ca+Mg) = 1.62–2.2:1; (K+Na):(Ca+Mg) = 1.9–2.1:1 9 [8,22]. Despite clearly defined thresholds for the nutrient ratios, some authors indicate that various plant species (grass, leguminous, herbaceous, crops) markedly differ in terms of their nutrient ratios [8,11,32,33,34]. The values of individual ratios given by the cited authors were varied, presenting both higher and lower values than those obtained in this study. An additional difficulty in the discussion of results is connected with a lack of proper references to the literature, in which there are no similar studies with the same plant species. In order for the considerations to be correct and credible, one should have the same point of reference, which in a given situation is not fully supported and good enough for comparison. Thus such differences in findings hinder the comparison and interpretation of data in this study.

In the presented study the biodiversity of plants did not play a significant role in this, because most of the nutrient ratio values calculated were very comparable, with one exception of K:Na ratio, where the values were higher for oat. The nutrient ratio values were shaped by immobilizing agents, which was particularly noticeable in the case of lupine. Generally, the obtained results of nutrient ratios for the plants from TI and TII were comparable and did not differ significantly. However, the influence of compost was more visible and led to slightly higher values of nutrient ratios. Jakubus and Bakinowska [8] also underlined the significant role of organic matter in the modification of quantitative nutrient ratios.

As previously indicated, from the perspective of agricultural practice, it is important to obtain the correct, optimal values of nutrient ratios. As shown by this study, they are difficult to obtain because the proper values were found only for Ca:Mg (2–3:1) and K:Mg (2–6:1). For Ca:Mg biomass ranged from 2.0 (TII) to 2.5 (TI) for lupine and from 2.5 (T0) to 3.7 (TI) for oat. The optimal values of K:Mg were obtained only in the case of plants cultivated on soil fertilized with compost, and they were 2.0 for lupine and 2.1 for oat. In the presented study, independently of the fact whether the immobilizing agent was applied or not, as well as regardless of the cultivated fodder plant, the values of K:Mg, K:(Ca+Mg) and (K+Na):(Ca+Mg) were below the expected range. In turn, the values of K:Na and Ca:P were significantly greater than the correct values, i.e., 5:1 (K:Na) and 2:1 (Ca:P). Also, [8] and [9] showed discrepancies between the expected value and the stated value. The authors explained those findings as well as the need for additional supplementation with mineral fertilizers [9] or the differences between plants in the intensity of nutrient uptake together with the consideration of the phenomenon of “luxury consumption” of K and antagonistic effect between macronutrients [8]. In this study, it is not possible to use such arguments because fertilization for plants has been balanced to meet their nutritional needs. Also, it is difficult to indicate a luxurious K uptake. According to obtained non-optimal values of nutrient ratios, K like P and Na appeared to be in insufficient amounts in plant biomass. Based on the obtained data, it can be concluded that the correct quantitative level in plants was reached only for Ca and Mg. Moreover, the antagonistic effect of K^+^ to Ca^2+^, Mg^2+^ and Na+ ions as well as the synergistic interaction of Ca and Mg should be considered in this interpretation. Feng et al. [33] stated that antagonistic or synergistic interactions among nutrients may occur during uptake from the soil and can influence the values of quantitative nutrient ratios in plant tissues. Moreover, the knowledge of nutrient interactions can guide the optimization of fertilization strategies for high yield and the efficiency of high nutrient use [13]. Such a natural behavior of nutrients should be accounted for in the interpretation of outcomes, especially with regard to antagonisms. The mentioned natural phenomena could cause meaningfully higher values of K:Na and Ca:P ratios and significantly lower values of K:Mg, K:(Ca+Mg) and (K+Na):(Ca+Mg).

According to Rietra et al. [13], Cu interacts with K, Mg and Ca, although the cited authors reported that the nature of such relationships is not clearly defined. This research seems to confirm this, especially for oat. This research confirmed the positive effect of Cu in soil fertilized with FA on the increased amount of K and Mg in plant biomass. In turn, the addition of compost to the soil contributed to an increase in Na and a decrease in K in oat along with an increase of Cu in the soil. The possibility of reduced accumulation of K and Mg in oat biomass as a result of soil contamination with Cu was indicated by De Conti et al. [35]. The authors also noted another relationship, which was not noted in this study, concerning the reduced concentration of P in shoots with increasing Cu levels in soil. The correlation between Cu and the analyzed nutrient ratios shows that, depending on the applied immobilizing agent, they were either negative (TI) or positive (TII) and were determined primarily for oats. Negative values of the correlation between Cu total in the soil fertilized with compost and the values of K:(Ca+Mg), K:Na, K:Mg and (K+Na):(Ca+Mg) allow us to assume that there is an active binding of Cu with the simultaneous release, especially of Ca and Mg. Such a possibility was indicated by Awasthi et al. [14].

## 5. Conclusions

The influence of compost and fly ash as immobilizing agents on nutrients and their mutual ratios was statistically proven. At the same time, they serve as sources of nutrients. The use of fly ash contributed to a higher content of elements in lupine and oat, while when the compost was used, higher values of nutrient ratios were obtained. Regardless of this, generally, the noticed differences between applied substances were not proved statistically, thus the obtained outcomes may confirm very comparable effects of both compost and fly ash. Therefore, compost or fly ash can be used alternatively in practice as immobilizing agents and the choice will only depend on their availability in the local market. It was found that the amounts of P, K and Na in plant biomass were too small, which should be interpreted by the antagonistic relations and the immobilizing effect of the substances used which was especially noticeable in the case of applied fly ash. Although the quantitative ratios allow a better assessment of the nutritional value of fodder plants, their practical use becomes debatable. This is evidenced not only by the literature data quoted in this study but also by the obtained results. The tested plants were properly fertilized, which met their nutritional needs, and they did not show any symptoms of a deficiency of macronutrients. However, no proper, optimal values of most macronutrient ratios were obtained. The exception was found for Ca:Mg and K:Mg. Regardless of the fodder plant and immobilizing agents used, Ca:Mg values were in the correct range of 2–3:1. The optimal values of K:Mg were obtained only for plants cultivated on soil fertilized with compost. Cu in soil indicates a possible nutritional imbalance of oat which was significantly modified by the immobilizing agents. Of course, the interpretation of the results admitted the possibility of antagonism between the nutrients, but this should not be considered the main cause. In the opinion of the authors, the knowledge of specific values of individual quantitative relationships between macronutrients should be revised. The correctness of the plant quality criteria used must be seen in a broader context, relating to the nutritional safety of animals and humans. This underlines the dependence in the flow of nutrients from soil through plants and animals to humans.

## Figures and Tables

**Table 1:** Basic properties of soil, compost and fly ash (data for composite samples) (after [19]).

Parameter	Soil	Compost	Fly Ash
pH	7.0	6.8	13.7
TOC (g·kg^−1^)	16.8	181.3	n.d. ^1^
P_Tot_ (g·kg^−1^)	1.8	3.8	10.8
K_Tot_ (g·kg^−1^)	2.9	3.5	3.7
Mg_Tot_ (g·kg^−1^)	3.5	1.5	8.4
Ca_Tot_ (g·kg^−1^)	13.4	18.4	18.4
Na_Tot_ (g·kg^−1^)	0.2	1.2	8.8
Cu_Tot_ (mg·kg^−1^)	200	33	44

^1^ n.d.—not determined.

**Table 2:** *p*-values from one-way ANOVA determined separately for each macronutrient in lupine and oat. Comparison between T0—control soil, TI—soil with compost addition and TII—soil with FA addition.

Macronutrient	Lupin	Oat
Ca	0.00825 **	0.00069 ***
K	7.57 × 10^−10^ ***	0.01371 *
Mg	0.00299 **	0.01281 *
Na	0.00159 **	0.66402
P	<2 × 10^−16^ ***	0.85213

Signif. codes: 0 ‘***’ 0.001 ‘**’ 0.01 ‘*’ 0.05 ‘.’ 0.1 ‘ ’ 1.

**Table 3:** *p*-values from one-way ANOVA determined separately for each nutrient ratios in lupine and oat. Comparison between T0, TI and TII.

Nutrient Ratios	Lupin	Oat
Ca:Mg	0.00053 ***	0.00076 ***
Ca:P	5.66 × 10^−8^ ***	0.00049 ***
K:(Ca+Mg)	0.01822 *	0.175
(K+Na):(Ca+Mg)	0.00841 **	0.162
K:Mg	0.00145 *	0.00019 ***
K:Na	0.271	0.423

Signif. codes: 0 ‘***’ 0.001 ‘**’ 0.01 ‘*’ 0.05 ‘.’ 0.1 ‘ ’ 1.

**Table 4:** Regression equations between Cu in soil (T0–TII) and the amount of macronutrients in oat and between Cu in soil and nutrient ratios in oat.

Treatment	Equation
TI	Na = 0.240 + 0.001·Cu
	K = 23.691 − 0.067·Cu
TII	K = −3.028 + 0.053·Cu
	Mg = −6.139 + 0.051·Cu
T0	K:Na = 120.545 − 0.509·Cu
TI	K:(Ca+Mg) = 1.353 − 0.004·Cu
	(K+Na):(Ca+Mg) = 1.375 − 0.004·Cu K:Mg = 5.320 − 0.015·Cu K:Na = 72.787 − 0.219·Cu
TII	K:(Ca+Mg) = −0.001 + 0.002·Cu
	(K+Na):(Ca+Mg) = 0.018 + 0.016·Cu
	K:Na = −3.736 + 0.117·Cu

## Data Availability

Not applicable.
